# Melasma secondary to drugs: a real-world pharmacovigilance study of the FDA adverse event reporting system (FAERS)

**DOI:** 10.1186/s40360-025-00912-4

**Published:** 2025-03-31

**Authors:** Yaxin Qu, Shuxin Wang, Hanzhang Xie, Xiao Meng, Bingnan Cui, Zhanshuo Xiao

**Affiliations:** 1 Department of Dermatology, Guang’anmen Hospital, China Academy of Chinese Medical Sciences, No. 5, Beixiange, Xicheng District, Beijing, 100053 China; 2 Beijing University of Chinese Medicine, Beijing, 100029 China

**Keywords:** Melasma, Adverse event, Ethinylestradiol and norethindrone, FAERS, Pharmacovigilance study

## Abstract

**Background:**

Melasma is a common hyperpigmentation disorder that causes significant distress to patients. In the real world, it is closely associated with various medications, making the timely identification and discontinuation of causative drugs an important aspect of clinical management. This study investigates the relationship between melasma and drug exposure based on data from the FDA Adverse Event Reporting System (FAERS) database.

**Methods:**

This study includes reports from the first quarter of 2004 to the second quarter of 2024, focusing on cases related to melasma. We employed four statistical methods to analyze the association between suspected drugs and adverse events related to melasma.

**Results:**

Within a specific timeframe, we extracted a total of 408 adverse reaction reports related to melasma. The result shows that a higher number of cases in female patients compared to male patients. The United States had the highest number of reported cases. We identified 22 drugs that were notably associated with melasma. Among these, the contraceptive “Ethinylestradiol and norethindrone” demonstrated the strongest signal of association.

**Conclusions:**

Melasma is associated with exposure to various medications, with a notable proportion of cases coincided with contraceptive use. The mechanisms involved include hormonal disturbances and oxidative stress.

## Introduction

Melasma is a common pigmentary disorder characterized by irregular, symmetrical brownish pigmentation on the face [1]. In certain populations, the prevalence can be as high as 50% [2]. Factors such as sun exposure, pregnancy, oral contraceptive use, genetic susceptibility, and the application of cosmetics are considered triggers for melasma [3]. The prevalence is significantly higher in women compared to men, and recurrence rates are also high [4]. Inappropriate activation of melanocytes and accumulation of melanin are important pathological mechanisms underlying the formation of melasma [5]. Patients with melasma are more likely to experience adverse emotional symptoms, significantly affecting their quality of life [6, 7]. Notably, oral medications such as contraceptives and phototoxic drugs are common triggers that can exacerbate melasma symptoms. Various medications can promote melanin synthesis, leading to the clinical manifestation of pigmented melasma [8]. Moreover, the treatment options for melasma are highly diverse, including oral medications, chemical peels, and physical therapies. Selecting the appropriate treatment method can be quite challenging [9], therefore, preventing the occurrence of lesions is of great significance. Therefore, systematically assessing the relationship between medications and melasma is important for the discovery and treatment of the condition.

Drug induction is an important cause of pigmented diseases and warrants our attention [10]. Hormones have been reported to have a close association with melasma. Studies have shown that the estrogen receptor levels are increased in the lesional skin of melasma compared to normal skin [11]. Furthermore, co-culture of melanocytes with estradiol promotes their proliferation [12], suggesting that hormone-related medications may be risk factors for melasma in certain contexts. Any other medications that may alter hormone levels also have the potential to adversely affect the development of melasma [13]. Additionally, the reactive oxygen species generated during melanin formation make melanocytes susceptible to oxidative stress, which plays an important role in the development of melasma [14]. Various medications or chemicals can induce redox imbalance within cells, leading to oxidative stress. The etiology of melasma is complex, so any medication that affects its underlying mechanisms deserves our attention.

However, due to the limitations in the duration of various studies, there has not been a comprehensive analysis of melasma resulting from adverse drug reactions. Our research aims to explore this relationship through a comprehensive analysis of drug-induced melasma using the FDA Adverse Event Reporting System (FAERS).

## Methods

This retrospective pharmacovigilance study is based on the FAERS database. FAERS is an adverse event reporting system established by the U.S. Food and Drug Administration (FDA) [15] designed to collect and analyze reports of adverse events and reactions associated with the use of drugs, vaccines, and medical devices in the market. Compared to long-term clinical trials, data mining from FAERS allows for a faster and more efficient evaluation of the relationship between drugs and adverse reactions, while also demonstrating high accuracy [16].

This research analyzed data from the first quarter of 2004 to the second quarter of 2024. This study utilized the preferred term for melasma from the Medical Dictionary for Regulatory Activities (MedDRA) and standardized it with various drug names from the DrugBank “Drugs” table, including brand names, generic names, synonyms, and abbreviations. All drugs were ultimately presented in their standardized generic name form.

The study employed four different analytical methods: Relative Odds Ratio (ROR: lower limit of 95% CI > 1, a ≥ 3), Positive Predictive Value (PRR: PRR ≥ 2, χ2 ≥ 4, a ≥ 3), Bayesian Classifier with Probabilistic Neural Network (BCPNN: IC025 > 0), and Elastic Band Greedy Method (EBGM: EBGM05 > 2) to assess the relationship between drugs and adverse events (melasma) in the FAERS database. All data obtained from these algorithms were statistically analyzed using R software (version 4.4.1).

## Results

**Fig. 1:**
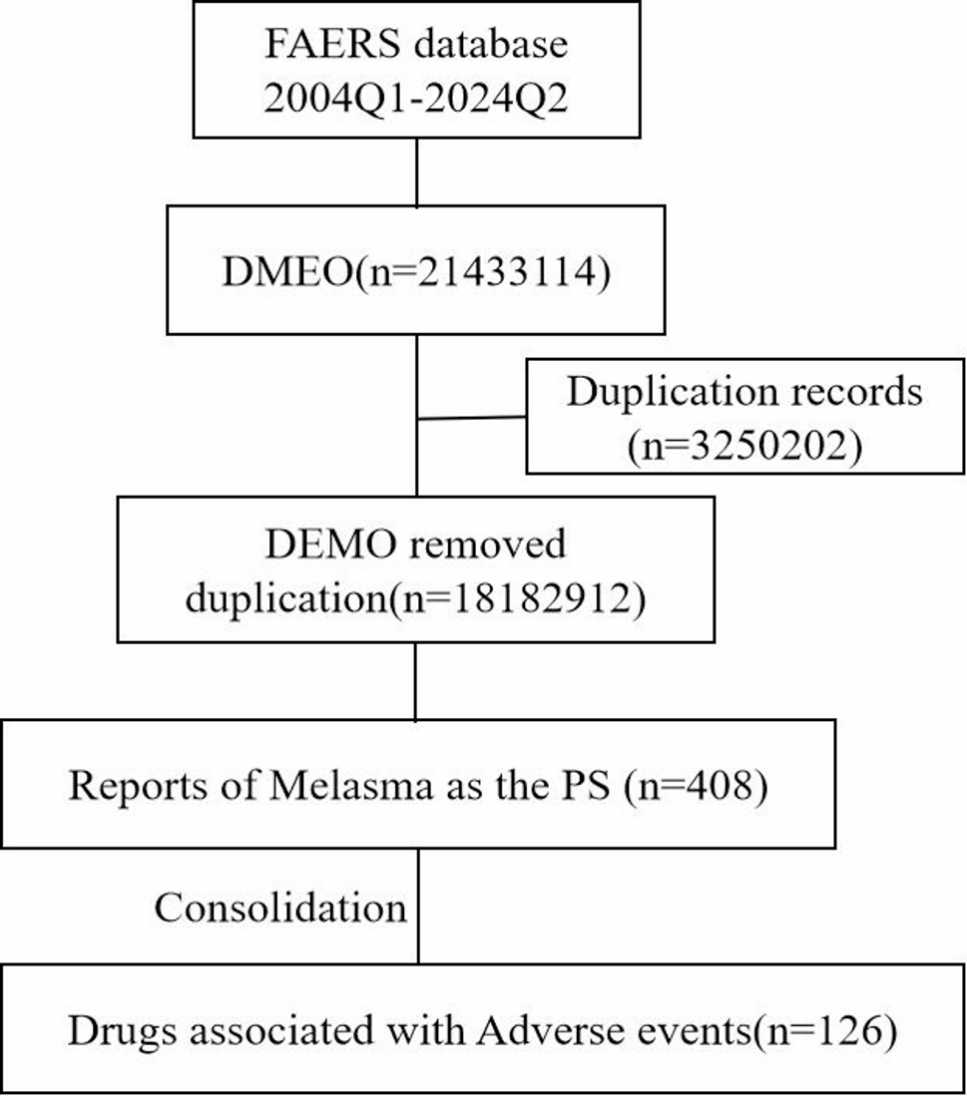
Flowchart for screening reports and drug targeting

**Fig. 2:**
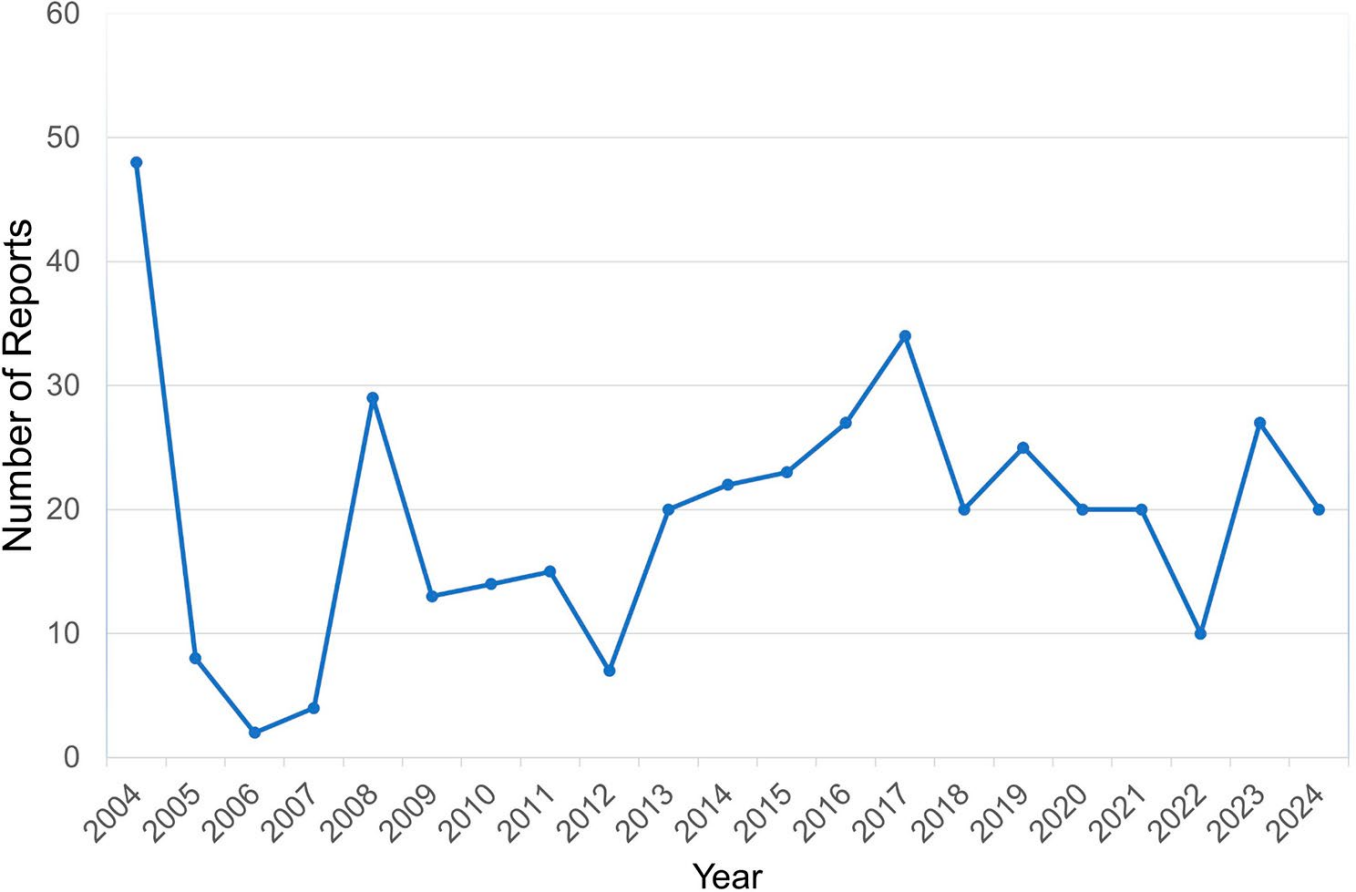
Annual distribution of reports associated with melasma collected from the FAERS database between 2004 and 2024

In this study, a total of 21,433,114 adverse event reports were extracted from the FAERS database. After removing duplicates, we obtained 18,182,912 unique adverse event reports. Among these, 408 reports were found to be associated with melasma. A flowchart of report screening and drug selection is provided in Fig. 1. The results indicate that the highest number of melasma-related adverse event reports occurred in 2004, with 48 reports, accounting for 11.8% of the total. Conversely, the fewest reports were recorded in 2006, with only 2. Since 2013, the number of adverse event reports related to melasma has stabilized annually. The annual statistics are shown in Fig. 2. Among all the included reports, females accounted for the majority, representing 84.8%, significantly higher than the 8.6% for males. Notably, 65.2% of the adverse event reports had missing weight information. The age group of 18–35 years represents the largest proportion in the report, accounting for 23.5%, while the age group of 51–65 and 66–85 years only accounts for 6.4% each. Among the reporters of adverse reactions, consumers made up 52.9%, followed by physicians at 19.1% and other health professionals at 10.0%. The country with the highest number of reports was the United States (57.4%), followed by the United Kingdom (5.6%) and France (4.7%). In terms of the outcomes of adverse reactions, death had the lowest proportion (0.5%), followed by life-threatening events (1.0%) and disability (3.7%). The most commonly reported indications were for contraception (27%), followed by acne, breast cancer, and multiple sclerosis. These basic statistics are summarized in Table 1.

**Table 1:** Demographic and clinical characteristics of melasma - related reports collected from the FAERS database between January 2004 and June 2024

Characteristics	Case number	Case proportion
**Number of events**	408	
**Sex**		
F	346	84.8%
M	35	8.6%
Missing	27	6.6%
**Weight(kg)**		
<50	12	2.9%
>100	7	1.7%
50~100	123	30.1%
Missing	266	65.2%
**Age(year)**		
18–35	96	23.5%
36–50	92	22.5%
51–65	26	6.4%
66–85	26	6.4%
Missing	168	41.2%
**Reported Person**		
Consumer	216	52.9%
Health Professional	18	4.4%
Lawyer	4	1.0%
Physician	78	19.1%
Other health-professional	41	10.0%
Pharmacist	12	2.9%
Missing	39	9.6%
**Reported Countries (top five)**		
United States	234	57.4%
United Kiongdom	23	5.6%
France	19	4.7%
Brazil	14	3.4%
Italy	9	2.2%
**Outcome**		
Death	2	0.5%
Life-Threatening	4	1.0%
Hospitalization	41	10.0%
Disability	15	3.7%
Other Serious	116	28.4%
Missing	227	55.6%
**Indications (top five)**		
Contraception	110	27.0%
Acne	8	2.0%
Breast cancer	8	2.0%
Multiple sclerosis	8	2.0%
Rheumatoid arthritis	7	1.7%

Among the 408 relevant reports, a total of 178 “primary suspect” drugs were identified. After consolidating and organizing the names of these drugs, we obtained a list of 126 distinct medications. Using various calculation methods, we identified 22 key drugs that met the criteria of all four disproportionality analysis methods, as shown in Table 2. Among these 22 drugs with significant associations, 12were hormonal agents, while others included antibiotics, targeted therapy drugs, and more. A detailed classification of these medications can be found in Table 3. The results from the four calculations indicate that “Ethinylestradiol and norethindrone” have the strongest association with melasma, with a ROR of 204.8, a PRR of 204.48, and an EBGM of 201.05. All four analysis methods ranked this combination at the top in terms of association strength. Following closely are “Ethinylestradiol and norelgestromin” and “Ethinylestradiol and levonorgestrel”, both of which are contraceptive drugs. This suggests a strong association between these medications and the occurrence of adverse events related to melasma. The number of reports related to “levonorgestrel” and melasma was the highest (*n* = 66), followed by “Ethynilestradiol and norelgestromin” (*n* = 44) and “Ethynilestradiol and drospirenone” (*n* = 34). This indicates a higher frequency of melasma occurrence among users of these drugs.

**Table 2:** Signal strength of drugs associated with melasma adverse reactions from the FAERS database (meeting criteria of all four disproportionality analysis methods)

DRUG	CaseNumbers	ROR(95%Cl)	PRR(χ^2^)	EBGM(EBGM05)	IC(IC025)
Ethinylestradiol and norethindrone	7	204.8(96.96-432.57)	204.48(1393.52)	201.05(107.55)	7.65(5.98)
Ethinylestradiol and norelgestromin	44	184.65(135.07-252.44)	184.42(7175.69)	164.97(126.99)	7.37(5.69)
Ethinylestradiol and levonorgestrel	10	148.94(79.5-279.03)	148.78(1432.49)	145.22(85.88)	7.18(5.51)
Ethinylestradiol and norgestimate	6	110.47(49.32-247.43)	110.38(640.95)	108.8(55.41)	6.77(5.09)
Ethinylestradiol and desogestrel	3	83.06(26.67-258.71)	83.01(241.32)	82.42(31.86)	6.36(4.69)
Conjugated estrogens and medroxyprogesterone acetate	6	42.61(19.03–95.43)	42.6(240.23)	42(21.39)	5.39(3.72)
Ethinylestradiol and drospirenone	34	35.78(25.19–50.81)	35.77(1054.91)	32.92(24.54)	5.04(3.37)
Encorafenib	3	27.76(8.91–86.43)	27.75(76.81)	27.56(10.65)	4.78(3.11)
Levonorgestrel	66	26.71(20.53–34.75)	26.71(1373.39)	22.62(18.15)	4.5(2.83)
Conjugated estrogens	5	16.3(6.75–39.38)	16.3(70.95)	16.12(7.71)	4.01(2.34)
Estradiol	10	15.9(8.49–29.77)	15.9(136.23)	15.54(9.19)	3.96(2.29)
Ethinylestradioland etonogestrel	7	14.24(6.75–30.06)	14.24(84.71)	14.02(7.5)	3.81(2.14)
Metronidazole	5	14.05(5.82–33.94)	14.05(59.88)	13.89(6.64)	3.8(2.12)
Clarithromycin	4	10.45(3.9-27.99)	10.45(33.87)	10.36(4.55)	3.37(1.7)
Finasteride	5	9.79(4.05–23.64)	9.79(38.97)	9.68(4.63)	3.28(1.6)
Medroxyprogesterone acetate	3	8.61(2.76–26.8)	8.61(20.02)	8.55(3.31)	3.1(1.42)
Isotretinoin	8	8.58(4.26–17.28)	8.58(52.57)	8.44(4.7)	3.08(1.4)
Anastrozole	3	7.44(2.39–23.17)	7.44(16.6)	7.39(2.86)	2.89(1.21)
Ribavirin	3	7.33(2.35–22.82)	7.33(16.28)	7.28(2.82)	2.86(1.19)
Paroxetine	8	6.49(3.22–13.06)	6.49(36.42)	6.38(3.55)	2.67(1)
Abemaciclib	5	6.49(2.68–15.66)	6.48(22.92)	6.42(3.07)	2.68(1.01)
Imatinib	7	5.92(2.8-12.49)	5.92(28.11)	5.83(3.12)	2.54(0.87)

**Table 3:** Categories of the top 22 drugs significantly associated with melasma and their anatomical therapeutic chemical (ATC) codes

DRUG	Drug categories	ATC codes
Ethinylestradiol and norethindrone	Sex hormones and modulators of the genital system	G03AB
Ethinylestradiol and norelgestromin	Sex hormones and modulators of the genital system	GO3AB
Ethinylestradiol and levonorgestrel	Sex hormones and modulators of the genital system	G03AB
Ethinylestradiol and norgestimate	Sex hormones and modulators of the genital system	G03AB
Ethinylestradiol and desogestrel	Sex hormones and modulators of the genital system	G03AB
Conjugated estrogens and medroxyprogesterone acetate	Sex hormones and modulators of the genital system	G03AB
Ethinylestradiol and drospirenone	Sex hormones and modulators of the genital system	G03AB
Encorafenib	Antineoplastic agents	L01EC
Levonorgestrel	Sex hormones and modulators of the genital system	G03FB
Conjugated estrogens	Sex hormones and modulators of the genital system	G03CC
Estradiol	Sex hormones and modulators of the genital system	G03CA
Ethinylestradioland etonogestrel	Sex hormones and modulators of the genital system	G03AB
Metronidazole	Antibacterials for systemic use	J01XD
Clarithromycin	Antibacterials for systemic use	J01FA
Finasteride	Urologicals	G04CB
Medroxyprogesterone acetate	Sex hormones and modulators of the genital system	G03FB/G03AC
Isotretinoin	Anti-acne preparations	D10BA
Anastrozole	Endocrine therapy	L02BG
Ribavirin	Antivirals for systemic use	J05AP
Paroxetine	Psychoanaleptics	N06AB
Abemaciclib	Antineoplastic agents	L01EF
Imatinib	Antineoplastic agents	L01EA

## Discussion

Melasma is a common dermatological disease. Analyzing the drugs associated with it can improve clinical diagnosis, treatment, and drug safety. Our study conducted a retrospective pharmacovigilance analysis of the FAERS database over the past 21 years, providing a comprehensive insight into this issue.

In our analysis, we found that female patients constituted the majority of adverse event (AE) reports associated with melasma, as observed in previous cross-sectional studies [17]. The higher incidence of adverse reactions in middle-aged and younger individuals may indicate that these events have an earlier age of onset. Notably, nearly half of the reporters identified themselves as consumers. However, due to the lack of specific information on whether these patients were taking medication under the direction of a physician, we cannot definitively conclude that there is a direct causal relationship between the occurrence of melasma and self-medication. This finding further supports the idea that certain drugs can trigger this adverse reaction. Our study also revealed that the highest number of reports originated from the United States, potentially linked to greater exposure to ultraviolet (UV) radiation [18] or a higher proportion of American women taking specific medications. Although there were few reports of fatal outcomes associated with melasma-related adverse reactions, a small risk of hospitalization and disability still remains. Contraception ranks first among the Indications, highlighting the significance of contraceptive drugs in this event. Overall, our analysis shows that hormone therapies are major risk factors for melasma, aligning with previous reports linking estrogen and progesterone to its onset. Clinical trials have demonstrated an association between oral contraceptives and the development of melasma [19, 20]. Furthermore, even during pregnancy, fluctuations in female hormones can lead to an increased incidence of melasma, although symptoms may improve postpartum [21]. This illustrates that both endogenous and exogenous hormones play a role in melasma formation. Estrogen stimulates the proliferation of melanocytes and endothelial cells, promoting keratinocytes to release pigment-stimulating factors such as endothelin-1 and stem cell factor (SCF), which result in localized hyperoxia and enhanced melanin production [22]. Progesterone can increase the number of melanocytes and enhances the activity of tyrosinase [23]. Antibiotics have also been linked to melasma [24], as their metabolism generates reactive oxygen species (ROS). The excessive production of ROS can alter the structure of tyrosinase, leading to liver damage, which may disrupt melanin metabolism and contribute to melasma development [25]. This explains the potential association between antibiotics and melasma. It is noteworthy that among the drugs we identified, Encorafenib is an antitumor agent used for melanoma that acts by inhibiting the BRAF gene [26]. A study has found that patients with BRAF gene mutations exhibited pigmented lesions [27]. However, their direct relationship requires further investigation. The analysis revealed that Anastrozole, a selective aromatase inhibitor used to treat estrogen-responsive breast cancer, may influence melasma development due to its effect on estrogen levels, given the well-established link between estrogen fluctuations and melasma [22]. Depression has also been reported to be associated with melasma [28]. The antidepressant Paroxetine, a selective serotonin reuptake inhibitor (SSRI), may modulate tyrosinase activity through serotonin receptors expressed in melanocytes. However, this mechanism is speculative and requires further investigation. Imatinib, a tyrosine kinase inhibitor, has been linked to cutaneous dyspigmentation in prior studies, potentially due to off target effects on c- KIT signaling, which regulates melanocyte survival and differentiation [29]. Although finasteride is primarily used to treat hair loss by inhibiting dihydrotestosterone, its main effects are focused on androgens. Nevertheless, there are documented cases linking its use to the development of melasma [30], suggesting it may indirectly influence the hormonal environment of the skin. Furthermore, Isotretinoin, a medication used to treat acne, is associated with hormonal imbalances [31]. The adverse effect of melasma may be linked not only to the medication but also to the underlying hormonal imbalance in acne. However, these observations derive from limited case reports and require more data for support.

In addition to estrogen imbalance and oxidative stress, there are other mechanisms related to the development of melasma that are worth noting. After UVB exposure, increased expression of SLC16A10 may induce melanin production by facilitating phenylalanine uptake. Keratinocytes release cytokines like α-MSH and EDN-1, which stimulate melanocyte activity [32], and dendritic cells produce more dendrites with increased melanosomes [33]. Studies have shown a remarkable relationship between zinc deficiency and melasma. Zinc is essential for skin health and cellular repair [34]. There is also a close relationship between thyroid autoimmune disorders and melasma in endocrine diseases [35]. This association may be attributed to the hormonal fluctuations and imbalances that accompany thyroid dysfunction. For instance, Hashimoto’s thyroiditis and Graves’ disease can lead to altered levels of thyroid hormones, which may influence melanocyte activity and result in increased melanin production [36]. Additionally, autoimmune disorders often involve systemic inflammation, which may exacerbate skin conditions like melasma. Understanding this connection highlights the importance of monitoring thyroid function in melasma patients, as improving thyroid health may alleviate skin pigmentation issues. It is important to note that genetic susceptibility is a significant factor contributing to the development of melasma [37]. Those with a genetic predisposition may be more sensitive to estrogen effects. Therefore, a comprehensive assessment is essential when evaluating medication-related adverse reactions in melasma.

Since a variety of medications can induce melasma, it is essential for patients taking high-risk drugs to adopt appropriate monitoring and preventive strategies. Regular skin assessments should be conducted for patients taking high-risk drugs. Additionally, protective measures, such as applying sunscreen, wearing wide-brimmed hats, and minimizing UV exposure, are necessary outdoors [38]. If the condition worsens, dose reduction or medication change may be considered following a doctor’s evaluation.

To summarize, it is evident that the drugs selected from the FAERS database are representative and encompass various mechanisms that contribute to the development of melasma. Currently, there is a wealth of analyses focusing on adverse reactions, while studies addressing disease-related issues are comparatively scarce. This research used real-world data to examine adverse events associated with melasma. Healthcare providers should be aware of these reactions when prescribing medications and to communicate them promptly to patients, thereby enhancing medication safety in clinical practice. However, our study has certain limitations. Firstly, the limited number of reports extracted from the database may affect the representativeness of the results. Additionally, factors like UV exposure, genetic predisposition, hormonal imbalances, and thyroid disorders [39] are key contributors to melasma development. These confounding factors can complicate the identification of drug-induced adverse events and, when combined, may worsen the condition. Nonetheless, our study did not comprehensively assess this condition. Future research will explore the role of genetic factors using genomic approaches and address the limitations of our current study. Additionally, this study lacks detailed statistics on individual factors such as the frequency of makeup application, number of pregnancies, and sunscreen usage. These factors are also relevant to the occurrence of melasma [40, 41]. This study may have limitations regarding missing treatment information. Many patients might be using multiple medications simultaneously, or combining certain drugs with other treatment regimens, and the FAERS database may not effectively capture drug interactions and their impact on melasma. Furthermore, the treatment duration and dosage of the target drugs were not considered, and these differences could also influence the results. The FAERS database also has certain limitations. Since FAERS is a voluntary reporting system, reports from different countries and healthcare professionals may be incomplete or inaccurate, which could introduce bias, leading to Reporting Bias. This bias may lead to an overrepresentation or underrepresentation of certain adverse events, affecting the generalizability and accuracy of the findings. Additionally, because it is not possible to distinguish whether an adverse event is caused by the medication or by the progression of the underlying disease itself, this may lead to Indication Bias during analysis. This may distort the true safety profile of the drug, leading to risks being either exaggerated or concealed. The absence of treatment duration and dosage information in FAERS limits our ability to assess dose dependent relationships or cumulative drug effects on melasma. For instance, prolonged use of hormonal contraceptives may exacerbate pigmentation, but this could not be quantified. Additionally, underreporting or incomplete data from certain regions may skew the observed geographic distribution of cases. In summary, despite certain limitations, this study is the first to analyze the comprehensive profile of melasma as an adverse event from a pharmacovigilance perspective, providing a valuable reference for healthcare professionals and contributing to the optimization of clinical practices.

## Conclusions

Our analysis of the FAERS database reveals that various drugs contribute to the development of melasma, highlighting a critical gap in the literature focused on disease-related issues. As the first comprehensive analysis of melasma from a pharmacovigilance perspective, our findings aim to guide clinical practice and enhance patient safety.

## Data Availability

No datasets were generated or analysed during the current study.
